# Trophic interactions of sharks and crocodylians with a sea cow (Sirenia) from the Miocene of Venezuela

**DOI:** 10.1080/02724634.2024.2381505

**Published:** 2024-08-28

**Authors:** Aldo Benites-Palomino, Gabriel Aguirre-Fernández, Jorge Velez-Juarbe, Jorge D. Carrillo-Briceño, Rodolfo Sánchez, Marcelo R. Sánchez-Villagra

**Affiliations:** 1 Department of Paleontology, University of Zurich, Karl-Schmid-Strasse 4, 8006 Zurich, Switzerland; 2 Department of Mammalogy, Natural History Museum of Los Angeles County, 900 Exposition Blvd., Los Angeles, California 90007, U.S.A.; 3 Museo Paleontológico de Urumaco, Urumaco, Estado Falcón, Venezuela; 4 Universidad Nacional Experimental Francisco de Miranda, Centro de Investigaciones Antropológicas Arqueológica y paleontológicas (CIAAP) Calle, Zamora Balcon Arcaya

## Abstract

Evidence of trophic interactions are not scarce in the fossil record, yet these are mostly represented by fragmentary fossils exhibiting marks of ambiguous significance. Differentiating between marks of active predation and scavenging events is therefore often challenging. Here, we report on a dugongine sea cow skeleton (partial skull and vertebrae) from the Lower to Middle Miocene Agua Clara Formation (Venezuela) with shark and crocodylian bite marks. The sirenian is identified as *Culebratherium* sp. and preserves crocodylian bite marks across the skeleton. The most conspicuous correspond to deep tooth impacts with dragging effect, concentrated in the rostrum of the specimen. We interpret these as the result of active predation because of the similarity with those produced when a crocodylian holds or rolls a prey. Additionally, shark bite marks can be observed throughout the skeleton, also evidenced by the finding of an isolated tiger shark (*Galeocerdo aduncus*) tooth associated with this skeleton. Because of the irregular distribution of the shark bite marks, these are interpreted as scavenging. Overall, these findings constitute one of the few records documenting multiple predators over a single prey, and as such provide a glimpse of the trophic networks during the Miocene in the region.

## INTRODUCTION

Evidence of trophic interactions between vertebrates is preserved in the fossil record mostly in the form of bite marks. Fossils of several groups of aquatic mammals such as pinnipeds or cetaceans have been found with bite marks in their bones at several localities across the globe (Benites-Palomino et al., 2022; Bianucci et al., 2010; Collareta et al., 2017; Godfrey & Altman, 2005). Such observations have a strong biological support as, for example, orcas and sharks have been recorded actively targeting toothed or baleen whales and pinnipeds in present-day oceans (Brown et al., 2010; Long & Jones, 1996). Despite this, the intrinsic limitations of the fossil record have complicated the assessment of further trophic interpretations as specimens with predation marks are often fragmentarily preserved.

Sirenians are the only extant group of herbivorous mammals to be fully adapted to an aquatic lifestyle in both marine and freshwater environments (Domning, 2018). Their modern diversity includes manatees (Trichechidae) and dugongs (Dugongidae). Adaptations such as the development of pachyosteoesclerotic bones and a thick layer of blubber make sirenians relatively slow swimmers (Domning & de Buffrénil, 1991), which in turn may suggest that sirenians are more vulnerable to predation than e.g., cetaceans. Yet only limited records of such events are known to paleontologists and neontologists as well. Observations in the wild have evidenced that different groups of predators hunt sirenians, with calves and juveniles being particularly at risk due to their smaller size. Modern Amazonian manatees might serve as occasional food for jaguars or caimans, especially during the dry season when water levels descend (Arraut et al., 2010; Reynolds III et al., 2009). Extant tiger sharks (*Galeocerdo*), orcas (*Orcinus*), crocodylians and hammerhead sharks (*Sphyrna*) have been recorded feeding on manatees and dugongs (Bonde et al., 2012; Vélez-Juarbe & Domning, 2014). Shark–sirenian interactions in the fossil record include, but are not restricted to, evidence of *Galeocerdo* feeding on a dugong from the Miocene of Austria (Feichtinger et al., 2022), a Miocene dugong from France with alleged shark bite marks (Rateau et al., 2009), a *Hexanchus*-sirenian interaction from the Pliocene of Italy (Merella et al., 2021), and a presumed scavenging case by small sharks on an Oligocene dugongid from South Carolina, U.S.A.

Here, we report on sirenian remains from the Lower to Middle Miocene Agua Clara Formation of northwestern Venezuela, southern Caribbean. This specimen consists of a fragmentary skeleton that includes a partial skull and 18 associated vertebrae. A series of marks detected all over the specimen are identified as shark and crocodylian bite marks. This specimen constitutes an exceptional case in the fossil record whereby preying evidence of not only one, but two different carnivores can be identified. Furthermore, this case of multiple interactions highlights the importance of sirenians within the Miocene trophic network of the Caribbean region which was once a hotspot of sirenian diversification during the Cenozoic (Domning, 2001).

## MATERIALS AND METHODS

**FIGURE 1.**
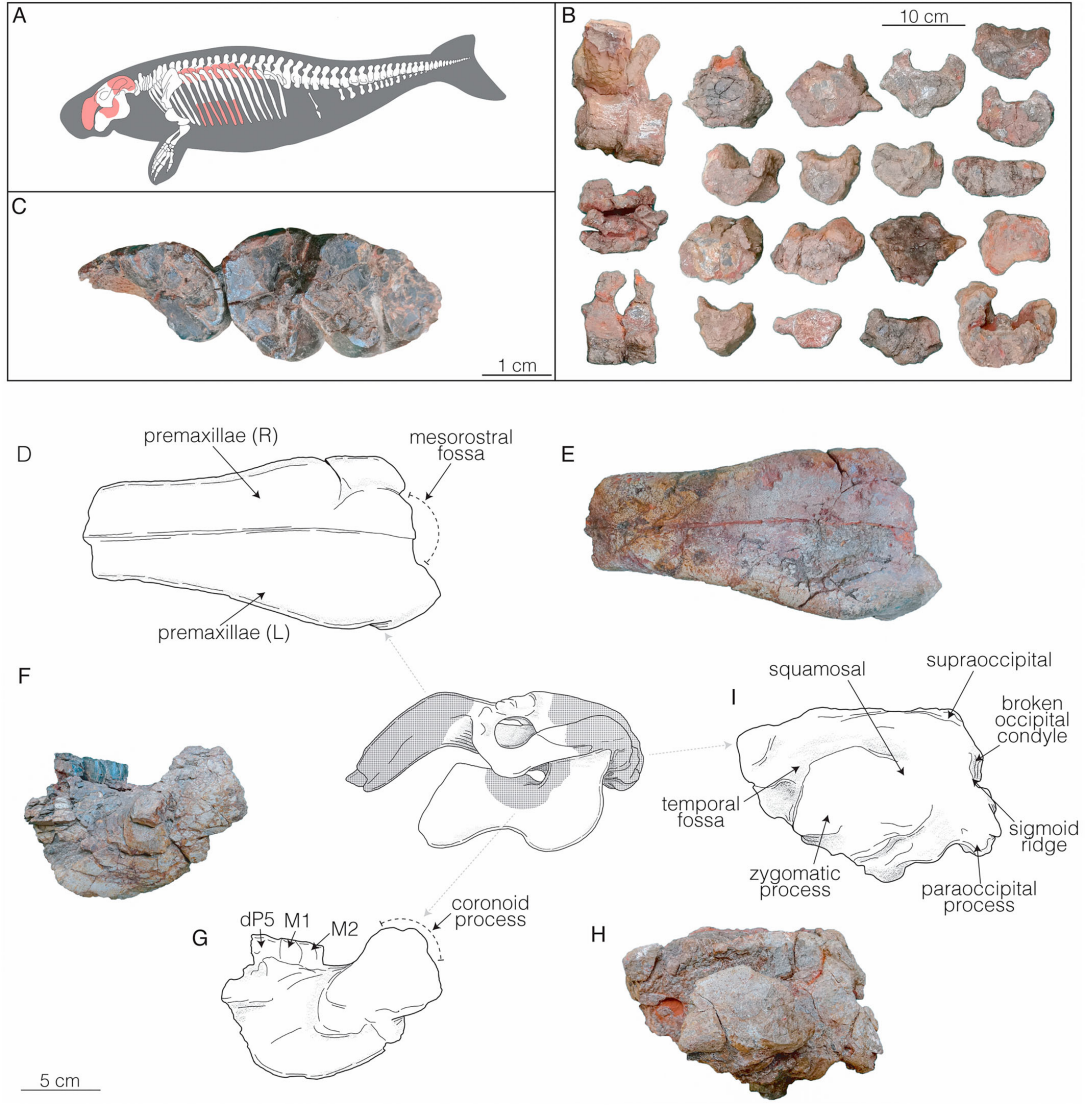
Agua Clara sirenian AMU-CURS-1248 remains consist of: **A**, **B**, a partial skeleton and associated skull fragments. **C**, detail view of the M2–M3 in occlusal view. **D**, **E**, skull fragments include the rostrum, **F**, **G**, dentary, and **H**, **I**, basicranium.

The dugongid specimen reported herein was collected from outcrops of the Agua Clara Formation (Fm.), south of the city of Coro, on the Coro-Churugura road, in the sector known as El Limón (11°21'25"N, 69°28'27"W). In the study area, the Agua Clara Fm. represents low energy environments and was deposited towards the end of a marine transgression. Based on foraminifera and invertebrates it has been assigned an Early Miocene age (Gamero, 1989; Quiroz et al., 2010). The studied specimen is represented by cranial and postcranial remains (Fig. 1) and was collected in 2019 by three of the authors (R.S., M.R.S.V, J.D.C.B) and other collaborators. These materials are permanently housed at the Museo Paleontológico de Urumaco, Falcón, Venezuela (AMU-CURS).

Only large, well-preserved bite marks were considered, as taphonomic processes in AMU-CURS-1248 have altered the preservation of the cortical bone across several parts of the skeleton. Three types of bite marks were recognized based on their shape, depth and (if recognizable) slashing orientation: (a) shallow round pits with a semi-circular outline, which might include some minor to moderate ruptures in the adjacent cortical bone; (b) wide, curved incisions with a rounder and deeper starting pit; (c) long and narrow slits with a triangular cross section.

## RESULTS

### Identification and Remarks on the Sirenian Remains

AMU-CURS-1248 is identified as a dugongine sirenian closely related to *Culebratherium alemani* based on the following combination of characters (Toledo, 1989; Velez-Juarbe & Wood, 2018): elongated premaxillary symphysis without a boss on its posterodorsal end, posterior border of the palatine deeply incised, pterygoid fossa, I1 greatly enlarged, with kite-shaped cross section, with the entire crown covered by enamel and high contact of the squamosals and parietals at the level of the temporal crest (Fig. 1D–H).

### Description and Identification of the Bite Marks

**FIGURE 2.**
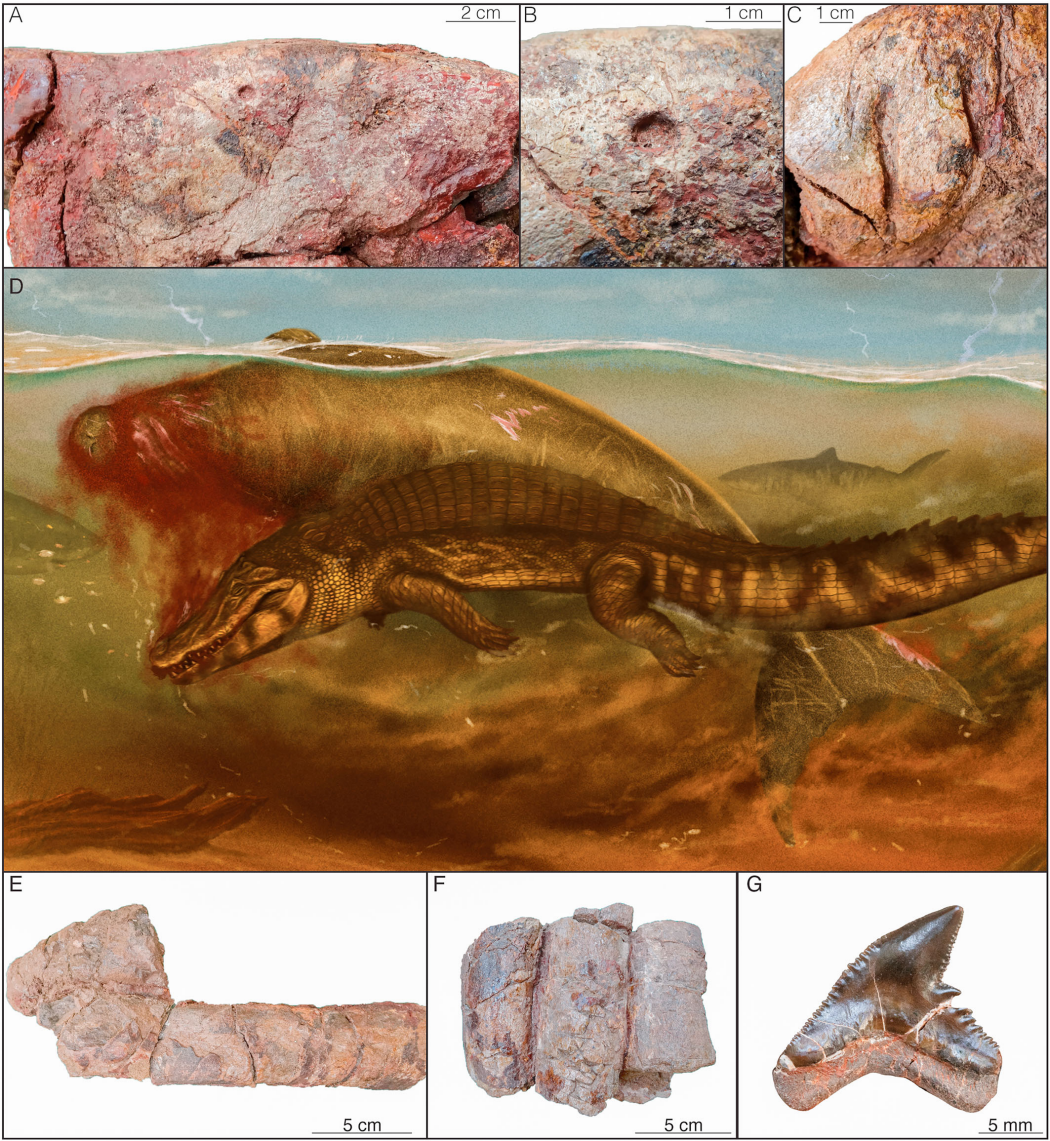
Bite marks on the Agua Clara sirenian AMU-CURS-1248. Crocodylian bite marks including: **A**, punctures; **B**, detailed view of A; and **C**, tearing marks. **D**, reconstruction of the trophic interactions by Jaime Bran. **E**, shark bite marks in the ribs, and **F**, detailed view. **G**, associated *Galeocerdo aduncus* tooth in labial view.

The first category of bite marks (group a) is only observed in the rostrum of AMU-CURS-1248 (Fig. 2A, B). Three punctures are preserved in the dorsal region of the premaxillae, anterior to the mesorostral fossa (Fig. S1). These pits reach a maximum width of 8 mm, with a semi-circular outline, except for two narrow lateral projections that result in a slightly bisected profile. Punctures with this round to bisected outline are identified as crocodylian bite marks (J. Njau & Gilbert, 2016; J. K. Njau & Blumenschine, 2006; Pujos & Salas-Gismondi, 2020). Due to the lack of additional traces (striation, slashing marks, hooks) the marks should have been produced by single biting events without further action.

The second category of bite marks (b) is derived from the first one and corresponds to marks that range from punctures with subsequent dragging effect, to wider curved incisions. These crocodylian marks are not easily distinguishable due to the fragmentary nature of the studied material, which locally includes the loss of the cortical bone (Fig. S2). Despite these preservation limitations, we identified two large incisions with a round starting impact and subsequent drag in the vicinity of the mesorostral fossa (Fig. 2C). This type of mark is only produced by biting events in which subsequent tearing, rolling, or grasping actions are executed (J. K. Njau & Blumenschine, 2006; Pujos & Salas-Gismondi, 2020).

The final group (c) is easily distinguishable from the two previous ones and includes long and narrow slits with V-shaped or triangular cross sections (Fig. 2E, F, and Fig. S3) which are typical bite marks produced by sharks (Jacobsen & Bromley, 2009; Muñiz et al., 2020). These marks are produced when the teeth impact the bone surface and are subsequently dragged during a series of biting events (Bianucci et al., 2010; Fulgosi, 1990). In AMU-CURS-1248 shark bite marks can be observed across the rostrum and zygomatic region of the skull, but also on the vertebrae and rib fragments (Fig. S3). The lack of serrations in the aforementioned marks indicate that these were not inflicted by otodontid sharks (with serrated teeth, e.g., *Otodus megalodon* or *Carcharodon carcharias*), but narrower teeth such as those of makos (e.g., *Isurus*) and/or tiger sharks (*Galeocerdo*). The shark identity is confirmed as *Galeocerdo aduncus* due to the discovery of an isolated tooth between the sirenian's neck and the thoracic ribcage.

## DISCUSSION AND CONCLUSIONS

In aquatic mammals such as cetaceans and pinnipeds, the record of trophic interactions has been gradually expanding (Benites-Palomino et al., 2022; Bianucci et al., 2010; Collareta et al., 2017; Ehret et al., 2009; Fulgosi, 1990), but the case of sirenians remains limited, with only few occurrences known so far (Feichtinger et al., 2022; Merella et al., 2021; Vélez-Juarbe & Domning, 2014). Present-day evidence has shown that crocodiles, orcas, and sharks prey on manatees and dugongs, mostly targeting calves and juvenile individuals as adults are difficult prey items because of their size (Anderson, 1985; Reeves et al., 1992; Wells et al., 1999). Yet, due to their fatty tissues, particularly the blubber, sirenians might constitute good food sources for predators. This tissue has a very high nutritional content and, because of its lipid-richness, can become the primary energy source of manatees during the dry season, which may extend as long as 200 days of fasting (Best, 1983; Iverson, 2009). The blubber, also present in other aquatic mammals such as baleen whales, is actively targeted by sharks, thus highlighting a clear feeding preference towards lipid-rich areas (Tucker et al., 2019).

The distribution of bite marks across the Agua Clara *Culebratherium* sp. indicates that it was preyed by crocodylians and sharks. Based on concentration of bite marks across the rostrum of the specimen (groups a & b), we hypothesize that the predator presumably tried to grasp the prey by the snout, maybe to suffocate the sirenian (Fig. S1). Such behavior is supported by the presence of marks with striations and slashing which might have been inflicted when a crocodylian executed a ‘death roll’ while grasping the prey, a common behavior of modern crocodylians. In the case of the shark bite marks (group c), their irregular distribution along with the variation in depth and orientation suggest that these belong to scavenging sharks, as reported for other aquatic mammals (Fig. S3). That said, and due to the fragmentary nature of the specimen, the possibilities of alternative scenarios cannot be ruled out.

The round pits with small lateral projections suggest that these were inflicted by a caimanine crocodilian of small to medium body size. The fossil site reported here is new, and vertebrate fossils from the Agua Clara Fm. were so far unknown, but caimanine remains from small to large-sized individuals have been found in other Miocene units of the Falcón basin such as the Socorro Fm and the lower member of the Urumaco Fm. (Scheyer et al., 2013). The presence of caimanines is also reported by the coeval strata of the Castilletes Fm. in Colombia (Jaramillo et al., 2020; Sánchez-Villagra, 2012), another indication of the widespread occurrence of these animals in the area (Moreno-Bernal et al., 2016). In the case of the shark bite-marks, the discovery of an isolated tooth of *Galeocerdo aduncus* (AMU-CURS-1364) reveals the likely tracemaker of most, if not all, the shark tooth marks. Similar bite-marks of tiger sharks over sirenians have been previously reported in the Styrian basin of Austria, and interpreted to belong to an active predation event due to the scarcity of *Galeocerdo* remains in the area, mostly due to the closed environment it represented (Feichtinger et al., 2022). Other instances of shark bite marks on dugongids have been documented, but not discussed in detail (Vélez-Juarbe & Domning, 2014).

Despite the fact that the Caribbean has been hypothesized as a great hotspot for sirenian diversification (Vélez-Juarbe & Domning, 2015; Velez-Juarbe & Wood, 2018), the fossil record of these marine mammals from Venezuela remains poorly known. Only a handful of remains have been reported from the area, mostly corresponding to fragmentary material and to the Late Miocene *Nanosiren sanchezi* (Domning & Aguilera, 2008). Nevertheless, the Miocene diversity of sirenians in the Caribbean is relatively low when compared with the greater diversity recorded in the Paleogene. However, given their much better Miocene record outside of the Caribbean region, this could be the result of collecting biases or scarcity of diagnostic material. Overall, the discovery of the Agua Clara dugongine not only expands the record of sirenians in the Southern Caribbean, but also provides a glimpse into the trophic relationships during the Early to Middle Miocene in the proto-Caribbean.

## Supplementary Material

Supplemental Material

## Data Availability

The authors confirm that the data supporting the findings of this study are available within the article or its supplementary materials.

## SUPPLEMENTARY FILES

Supplementary File 1.docx: Systematic Paleontology, including Figs. S1, S2, and S3.
