# Supplementary material for: Trophic interactions of sharks and crocodylians with a sea cow (Sirenia) from the Miocene of Venezuela
Source: J Vertebr Paleontol. 2024 Aug 28;43(6):e2381505. doi: 10.1080/02724634.2024.2381505 (PMC11824499; doi:10.1080/02724634.2024.2381505)
Supplement: Supplemental Material [file UJVP_A_2381505_SM2236.zip › Benites_SupplementR-HM.docx]

Supplementary Information

Trophic interactions of sharks and crocodylians with a sea cow (Sirenia) from the Miocene of Venezuela

ALDO BENITES-PALOMINO^1^,^*^ GABRIEL AGUIRRE-FERNÁNDEZ^1^, JORGE VELEZ-JUARBE^2^, JORGE D. CARRILLO-BRICEÑO^1^, RODOLFO SÁNCHEZ^3,4^ & MARCELO R. SÁNCHEZ-VILLAGRA^1^

^1^Department of Paleontology, University of Zurich, Karl-Schmid-Strasse 4, 8006 Zurich, Switzerland, aldo.benitespalomino@uzh.ch;

^2^Department of Mammalogy, Natural History Museum of Los Angeles County, 900 Exposition Blvd., Los Angeles, California 90007, U.S.A.

^3^Museo Paleontológico de Urumaco, Urumaco, Estado Falcón, Venezuela

^4^ Universidad Nacional Experimental Francisco de Miranda, Centro de Investigaciones Antropológicas Arqueológica y paleontológicas (CIAAP) Calle, Zamora Balcon Arcaya.

**Content**

- Systematic Palaeontology
- Figure S1.
- Figure S2.
- Figure S3.

**Systematic Palaeontology**

MAMMALIA Linnaeus, 1758

SIRENIA Illiger, 1811

DUGONGIDAE Gray, 1821

DUGONGINAE Gray, 1821 *sensu* Vélez-Juarbe and Wood et al. 2019

*Culebratherium* Vélez-Juarbe and Wood, 2019

*Culebratherium* sp.

(Figs. S1, S2, and S3)

Referred Specimen: AMU-CURS-1248 a partly preserved skull including premaxillae, fragmentary maxillae, the right squamosal, occipital region, and mandible, plus eighteen fragmentary vertebrae and associated rib fragments.

Locality and Horizon: The dugongid specimen here reported was collected from outcrops of the Agua Clara Formation near the Coro-Churugura road (11°21'25"N, 69°28'27"W). The Agua Clara Fm. in the area of study represents marine low-energy environments, deposited towards the end of a marine transgression, with an early Miocene age [4,5].

Description and remarks: The preserved portion of the rostrum of AMU-CURS-1248 displays a flat to gently convex surface and has a trapezoidal outline, being slender towards the distal end and broadening towards the anterior wall of the mesorostral fossa. Ventrally, the palatal surface is eroded, but the premaxillary suture can be distinguished. The incisor alveoli are enlarged, with a depth that extends more than half of the length of the premaxillary symphysis. The I1 are not fully erupted and would have not stuck out much beyond the gum. In anterior view, both incisors are mediolaterally compressed, with a lozenge or kite-shaped cross-section, preserving an enamelled-cap. The cranial fragment is dorsally flat; however, it is not clear is this is product of the taphonomic process or a feature of the skull. In dorsal view, only the left frontal pit can be distinguished, having a semi-circular outline and being shallow as in *Culebratherium alemani* [6]. The fronto-parietal suture is not clear due to poor preservation of the cortical bone, but it was likely ‘v’ or ‘u’ -shaped as in other dugongids. The temporal crests in the parietal are prominent, becoming transversely wider posteriorly, reaching their widest mediolateral extension at the level of their respective squamosal, before diverging anterolaterally, resembling the temporal crest observed in some specimens of *Metaxytherium floridanum* [7]. The left squamosal is robust, slightly projecting dorsally at the base of the zygomatic arch. In ventral view, only a small portion of the glenoid fossa can be distinguishable, being shallow and with an elliptical profile. Only the dorsoposterior region of the exoccipital is preserved, impeding to recognise the shape of the foramen magnum nor the extension/depth of the supracondylar fossa.

Although fragmentary, AMU-CURS-1248 can be referred the genus *Culebratherium* based on the broad rostrum, lack of a boss at the posterodorsal end of the premaxillary symphysis, enlarged tusk incisors with a kite-shaped cross section, and flat frontal skull roof. These characters distinguish *Culebratherium alemani* and closely related taxa that are currently classified as *Dioplotherium* (i.e., D. *allisoni*, and *Dioplotherium* cf. *D. allisoni;* see Velez-Juarbe and Wood, 2019:fig. 8), but that should be considered as belonging to a more inclusive *Culebratherium*, while *Dioplotherium* should be restricted to *Dioplotherium manigualti*.

**Supplemental information references**

1. Linnaeus C. 1758 Systema Naturae, edition X, vol. 1 (Systema naturae per regna tria naturae, secundum classes, ordines, genera, species, cum characteribus, differentiis, synonymis, locis. Tomus I. Editio decima, reformata). *Holmiae Salvii* 1, 824.

2. Illiger JKW. 1811 *Caroli Illigeri... Prodromus systematis mammalium et avium: additis terminis zoographicis utriusque classis, eorumque versione germanica*. sumptibus C. Salfeld.

3. Gray JE. 1821 On the natural arrangement of vertebrate mammals. *Lond. Med. Repos.,* 15, 296–310.

4. Gamero MD de. 1989 El Mioceno temprano y medio de Falcón septentrional. *GEOS Rev. Venez. Cienc. Tierra*, 25–35.

5. Quiroz L, Jaramillo C, Sánchez-Villagra M, Aguilera O, Carlini A. 2010 Stratigraphy and sedimentary environments of Miocene shallow to marginal marine deposits in the Urumaco Trough, Falcon Basin, western Venezuela. *Urumaco Venezuelan Paleontol*., 153–172.

6. Velez-Juarbe J, Wood AR. 2019 An early Miocene dugongine (Sirenia: Dugongidae) from Panama. *J. Vertebr. Paleontol.* 38, e1511799. (doi:10.1080/02724634.2018.1511799)

7. Domning DP. 1988 Fossil Sirenia of the West Atlantic and Caribbean region. I. *Metaxytherium floridanum* Hay, 1922. *J. Vertebr. Paleontol.,8, 395*–*426.*  (doi:10.1080/02724634.1988.10011728)


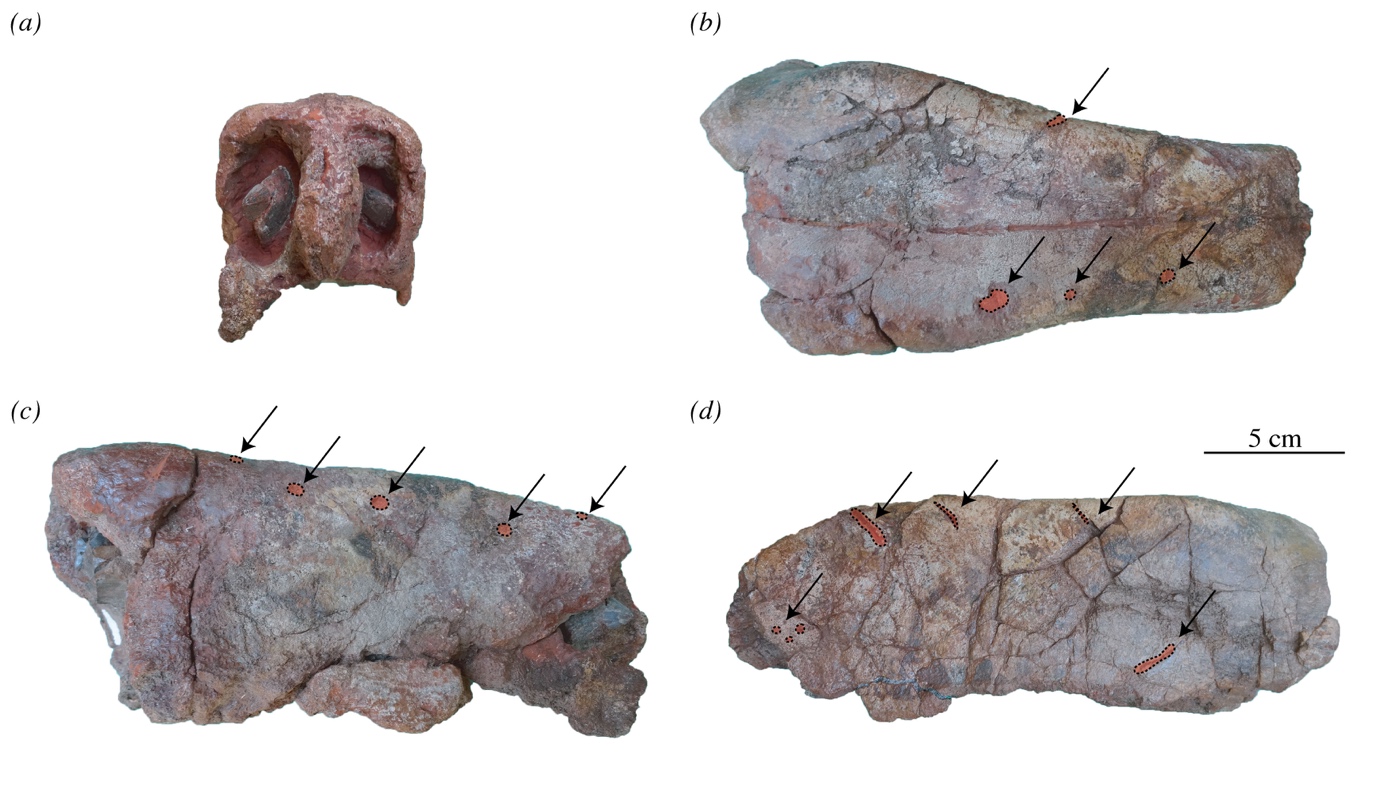


**Figure S1**. Agua Clara sirenian AMU-CURS-1248 rostrum fragment in anterior (a), dorsal (b), right lateral (c) and left lateral (d) views highlighting the shark bite marks & crocodilian tooth impacts.


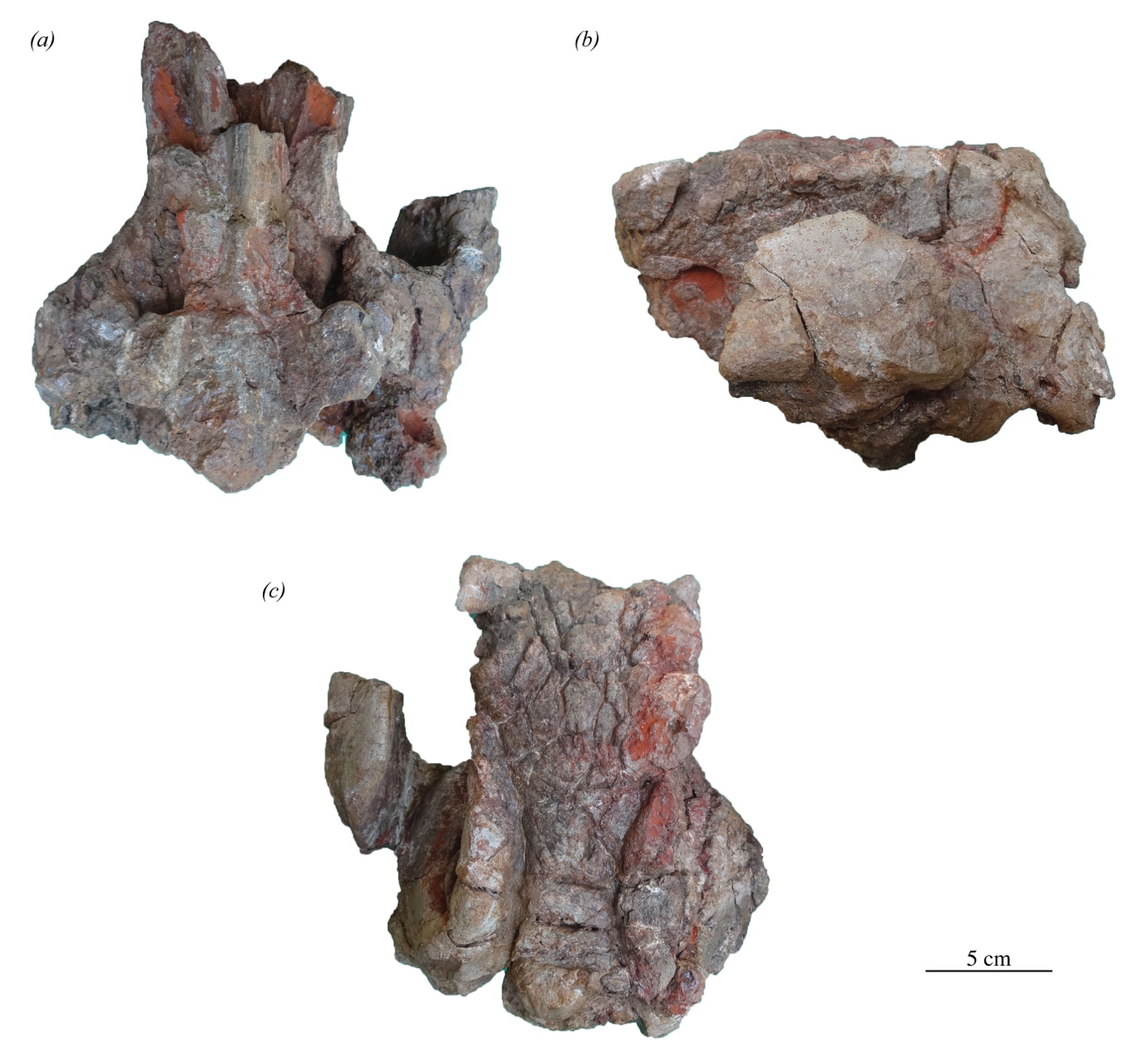


**Figure S2**. Agua Clara sirenian AMU-CURS-1248 braincase in ventral (a), left lateral (b), and dorsal (c) views.


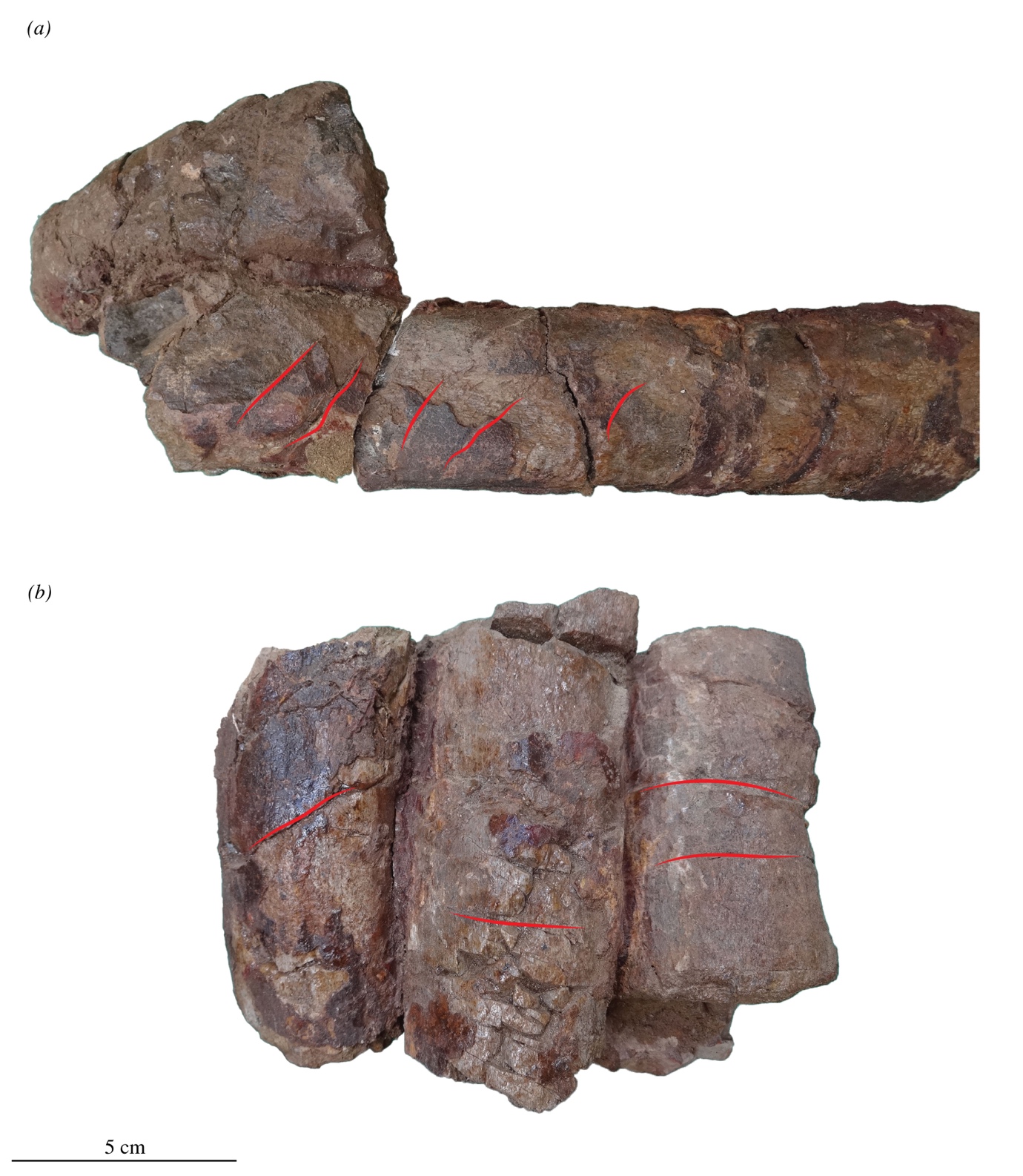


**Figure S3**. Agua Clara sirenian AMU-CURS-1248 rib fragments highlighting the shark bite marks (a & b) in lateral views.
